# Childhood cognitive ability and the risk of self-harm and suicide in later life.

**DOI:** 10.23889/ijpds.v7i3.2052

**Published:** 2022-08-25

**Authors:** Matthew Iveson

**Affiliations:** 1 The University of Edinburgh

## Objectives

Suicide rates are high among older adults, with self-harm as an important risk factor. In middle-aged adults, self-harm and suicide risk appears to be predicted by early-life factors including cognitive ability. The present study examines whether associations between early-life factors and self-harm and suicide can be observed among older adults.

## Approach

We construct a large, representative cohort using participants of the Scottish Mental Survey 1947 – a nationwide assessment of cognitive ability and socioeconomic conditions administered to all 11-year-olds attending a Scottish school (N ~ 70,000). We link research data from childhood to later-life (age 50+) routinely-collected hospital admissions and deaths data.

## Results

Using survival analyses, we report the associations between early-life predictors – including childhood cognitive ability – and the risk of self-harm and suicide in later-life, further adjusting for proximal socioeconomic conditions and comorbidities.

## Conclusion

We demonstrate the importance of early-life factors for predicting self-harm and suicide among older adults, highlighting potential mechanisms, modifiable factors and markers. The implications of the results for research and policy are discussed.

